# Ecological plasticity and commercial impact of invasive marbled crayfish populations in Madagascar

**DOI:** 10.1186/s12898-019-0224-1

**Published:** 2019-02-06

**Authors:** Ranja Andriantsoa, Sina Tönges, Jörn Panteleit, Kathrin Theissinger, Vitor Coutinho Carneiro, Jeanne Rasamy, Frank Lyko

**Affiliations:** 1 Division of Epigenetics, DKFZ-ZMBH Alliance, German Cancer Research Center (DKFZ), Im Neuenheimer Feld 580, 69120 Heidelberg, Germany; 2 Institute for Environmental Sciences, University of Koblenz-Landau, Fortstrasse 7, 76829 Landau, Germany; 3 Mention Zoologie et Biodiversité Animale, Université d’Antananarivo, BP906, 101 Antananarivo, Madagascar

**Keywords:** Marbled crayfish, Madagascar, Ecology, Habitat diversity, Crayfish plague, Farming

## Abstract

**Background:**

The marbled crayfish (*Procambarus virginalis*) is a monoclonal, parthenogenetically reproducing freshwater crayfish species that has formed multiple stable populations worldwide. Madagascar hosts a particularly large and rapidly expanding colony of marbled crayfish in a unique environment characterized by a very high degree of ecological diversity.

**Results:**

Here we provide a detailed characterization of five marbled crayfish populations in Madagascar and their habitats. Our data show that the animals can tolerate a wide range of ecological parameters, consistent with their invasive potential. While we detected marbled crayfish in sympatry with endemic crayfish species, we found no evidence for the transmission of the crayfish plague pathogen, a potentially devastating oomycete. Furthermore, our results also suggest that marbled crayfish are active predators of the freshwater snails that function as intermediate hosts for human schistosomiasis. Finally, we document fishing, farming and market sales of marbled crayfish in Madagascar.

**Conclusions:**

Our results provide a paradigm for the complex network of factors that promotes the invasive spread of marbled crayfish. The commercial value of the animals is likely to result in further anthropogenic distribution.

**Electronic supplementary material:**

The online version of this article (10.1186/s12898-019-0224-1) contains supplementary material, which is available to authorized users.

## Background

The marbled crayfish *Procambarus virginalis* [1] is the only known decapod crustacean that reproduces by obligate parthenogenesis [2]. While the precise origin of the animals remains to be identified, the first record of marbled crayfish is from the German aquarium trade in 1995 [1, 2], indicating a very recent evolutionary origin. Based on morphological characters and genetic data, the sexually reproducing slough crayfish from Florida, *Procambarus fallax*, has been identified as the most closely related species [2, 3]. Additional evidence strongly suggests that marbled crayfish separated from *P. fallax* by major genetic changes [4, 5], which may have occurred as recently as 25 years ago. The combination of obligate parthenogenesis and very young evolutionary age has generated a population that can be considered a single genetic clone [6–8].

According to the Red Queen hypothesis [9], lack of genetic variation severely curtails the ability of a species to adapt and proliferate [10]. However, the genetically homogeneous marbled crayfish has been described as a successful invasive species in various countries [11–18]. This is exemplified by the situation in Madagascar, where marbled crayfish were first introduced around 2005 [11]. By 2008, the animals had already spread considerably and became widely recognized in the area around the capital city Antananarivo [11, 12]. In 2017, marbled crayfish had colonized an area of approximately 100,000 km^2^, stretching from the highland to the coast [8]. This area includes several habitats that are not inhabited by the relatively narrowly distributed native crayfish species of Madagascar [19, 20].

The invasiveness of marbled crayfish represents a key feature to define their overall impact [13, 21]. However, additional factors can contribute to this picture. For example, marbled crayfish may transmit the crayfish plague agent [22], the oomycete *Aphanomyces astaci*, which has eradicated major crayfish populations in Europe [23]. On the other hand, freshwater crayfish can also function as biocontrol agents for human diseases, such as schistosomiasis [24]. This is exemplified by their effects on the snail populations that function as intermediate hosts for *Schistosoma* flatworms and includes the direct predation of the snails and the consumption of aquatic plants that are used by the snails for shelter, as oviposition sites and as food [25]. Lastly, freshwater crayfish also represent an increasingly important source of nutritional protein for human consumption [26].

Despite their invasive spread, habitats of marbled crayfish have not been analyzed systematically yet. Madagascar is characterized by a high climate and habitat diversity. The broad range of aquatic ecosystems renders the island ideally suited to better understand marbled crayfish ecology. Furthermore, Madagascar hosts a unique and diverse flora and fauna, including seven endemic crayfish species from the genus *Astacoides* [19], which are potentially threatened by crayfish plague outbreaks. Finally, marbled crayfish have been spreading in Madagascar for the past 10 years [8], but their potential for human use has not been elucidated. Our study aims to provide a detailed description of the large population of marbled crayfish in Madagascar. Our results shed light on the animals’ ability to colonize new environments, their impact on local freshwater ecosystems and their increasing commercial importance.

## Results

**Fig. 1:**
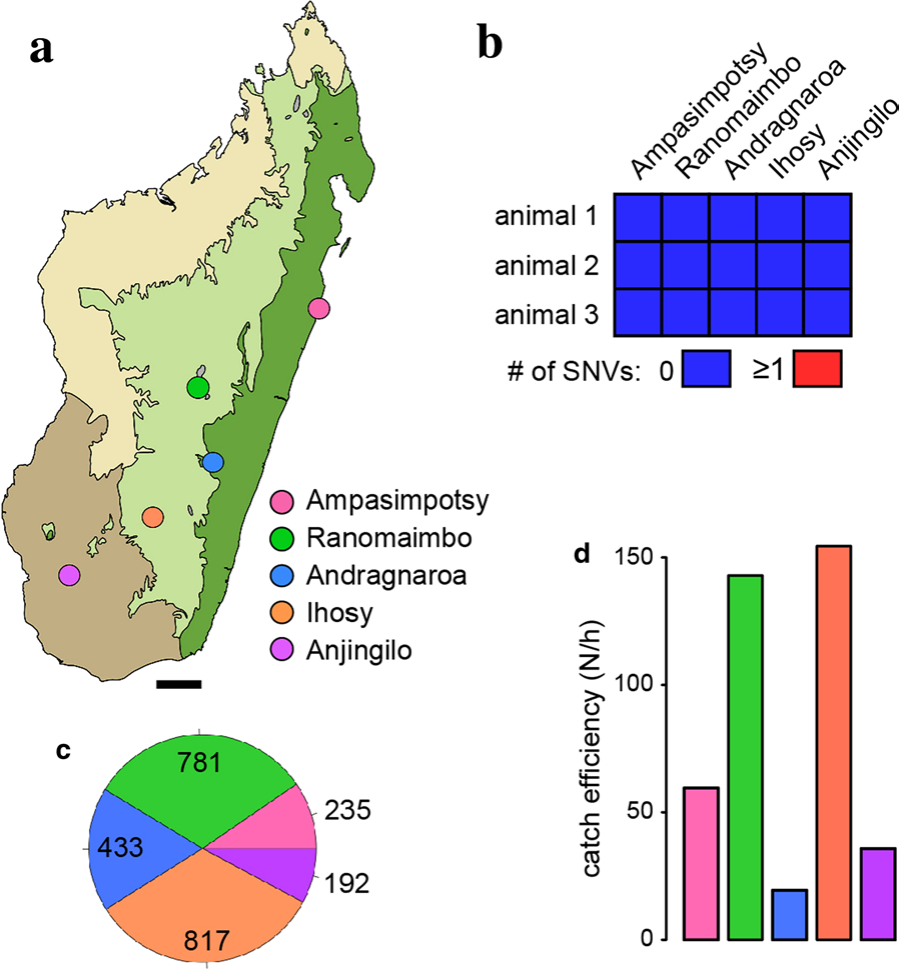
Overview of the study. **a** Map of Madagascar (generated by the first author) indicating the location of the 5 selected sites. Bar: 100 km. Area colors indicate bioclimates, see “Methods” for details. Dark green: humid, light green: sub-humid, grey: montane, beige: dry, brown: sub-arid. **b** Genetic authentication of 3 randomly selected animals per site. The number of single-nucleotide variants in the mitochondrial cytochrome B gene was 0 for all animals tested. **c** Numbers of animals that were analyzed at each site. **d** Catch efficiencies (number of catches for 2 persons per hour) for each site

After an initial field survey that determined the distribution area of marbled crayfish in Madagascar [8], we performed a more detailed follow-up analysis. We investigated five aquatic ecosystems consisting of three lentic and two lotic environments in four out of the five bioclimatic zones of Madagascar (Fig. 1a, Additional file 1). The lentic ecosystems include a randomly selected pond in the middle of a village on the east coast (Ampasimpotsy), a lake on the highlands in the center of a big city impacted by human activities (Ranomaimbo) and a rice field (Anjingilo) in a relatively isolated area in the south. Furthermore, two lotic habitats were included: a slow-flowing highland river located in the Ranomafana National Park rainforest (Andragnaroa) and a slow-flowing lowland river located near a large city in south-central Madagascar (Ihosy). Sequencing of PCR amplicons from three randomly collected animals from each of the five sites showed complete identity with the marbled crayfish reference sequence (Fig. 1b), and thus provided genetic authentication for the populations analyzed in this study.

**Fig. 2:**
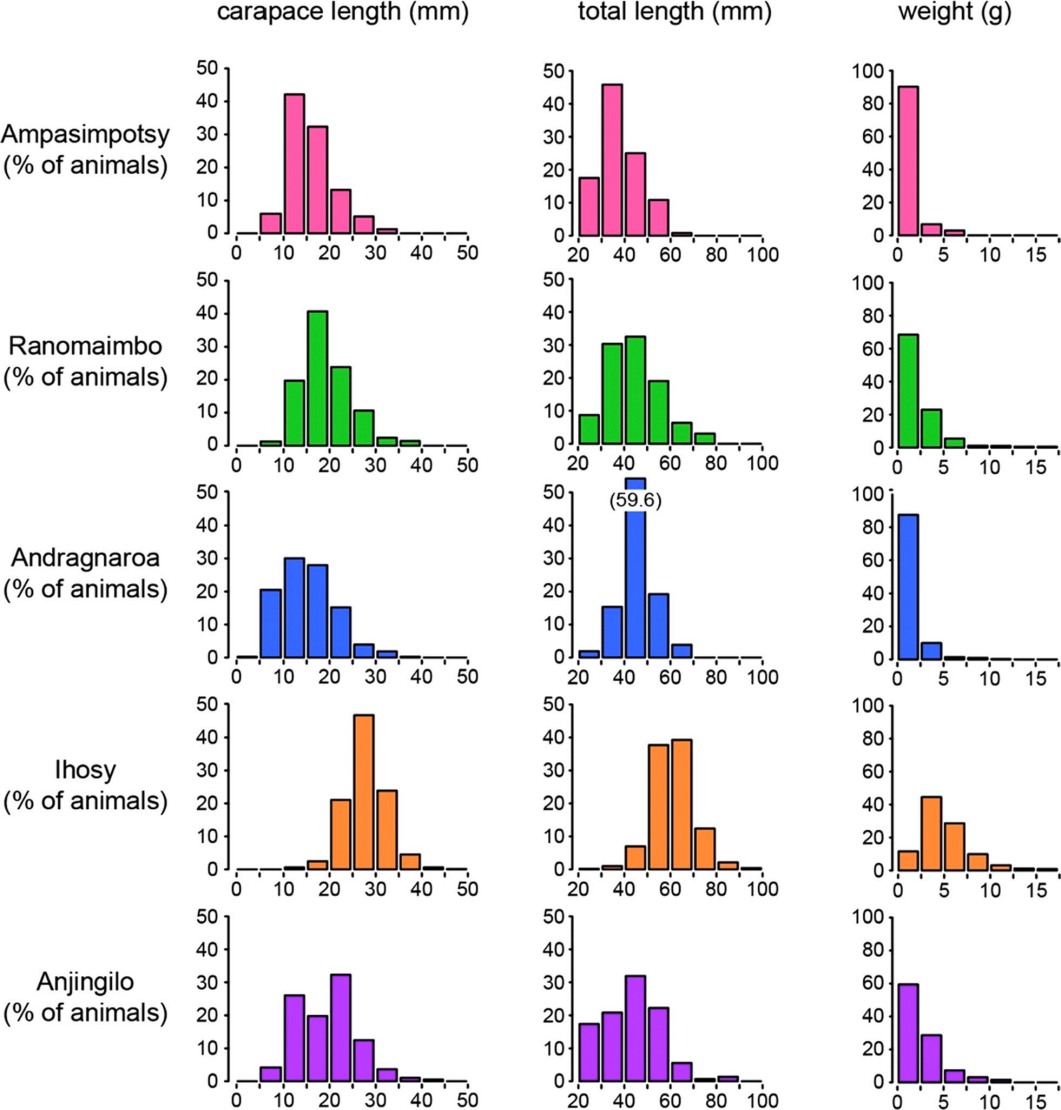
Population structures at different locations. Distribution of carapace lengths, total lengths and weights of marbled crayfish in the Ampasimpotsy pond, the Ranomaimbo lake, the Andragnaroa river, the Ihosy river and the Anjingilo rice field

In total, we collected 6641 crayfishes and obtained data for 2458 animals (192–817 animals per site, Fig. 1c). Catch per hour results differed considerably between individual sites, ranging from 154 animals in Ihosy to < 20 animals in Andragnaroa (Fig. 1d). These results suggest that population densities differ between the analyzed sites. Measurements established carapace lengths that were often between 10 and 35 mm and total lengths between 30 and 80 mm (Fig. 2). Animal weights usually ranged from 1 to 10 g (Fig. 2). Marbled crayfish were significantly (p < 0.05, Kruskal–Wallis one-way analysis of variance) larger and heavier in the Ihosy river as compared to the remaining sites (Fig. 2). The reasons for these differences remain to be established.

**Fig. 3:**
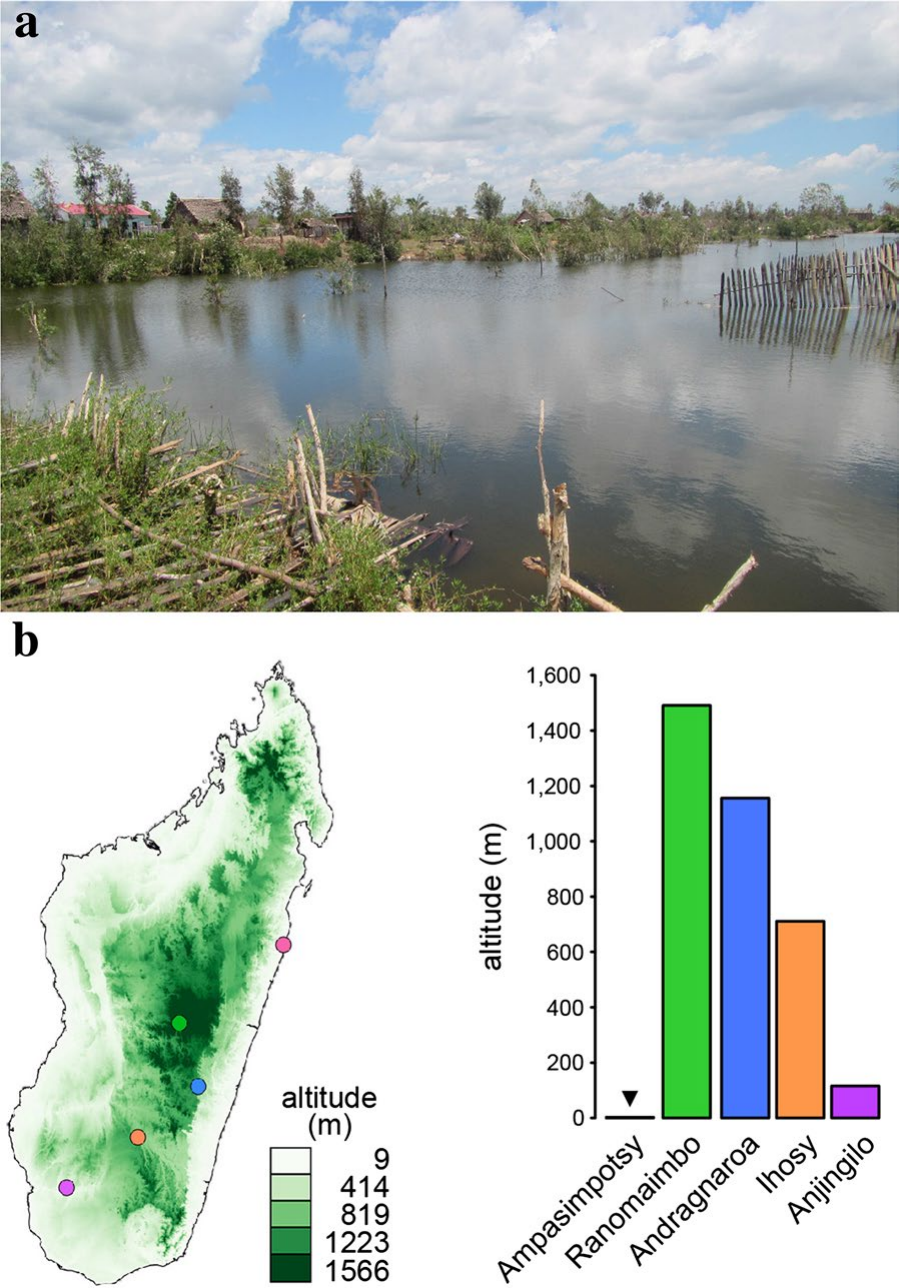
Marbled crayfish populations inhabit ecological niches at different altitudes. **a** Picture of the Ampasimpotsy coastal pond, which is located 3 m above sea level. **b** Map of altitudinal gradients of Madagascar and altitudes of the 5 selected sites. Picture and map were produced by the first author

**Fig. 4:**
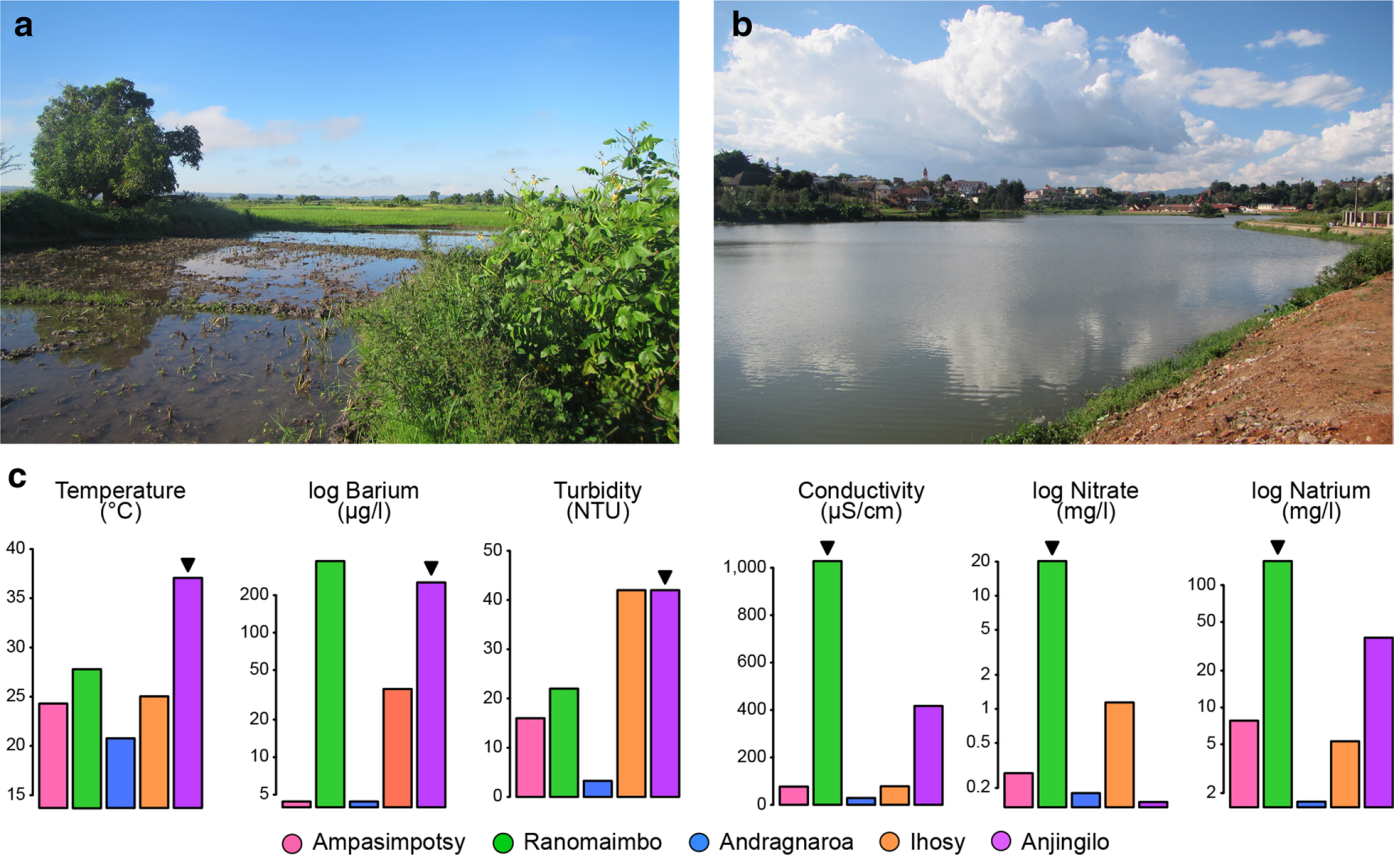
Marbled crayfish population habitats. **a** Picture of the Anjingilo rice field and **b** the Ranomaimbo lake. **c** Specific water parameters of the five habitats analyzed. All pictures were produced by the first author

Subsequent analyses revealed remarkable differences for several fundamental ecological parameters. For example, while marbled crayfish were initially described to inhabit the central highland of Madagascar [11], the population in the Ampasimpotsy pond was located almost at sea level (Fig. 3a). In fact, we detected marbled crayfish at a wide range of altitudes (3–1491 m, Fig. 3b). Additional examples for the ability of marbled crayfish to colonize different habitats were provided by the Anjingilo rice fields (Fig. 4a) that are irrigated by thermal water and the Ranomaimbo lake located in the city center of Antsirabe (Fig. 4b). The water of Anjinglio is characterized by a particularly high temperature of 37 °C and elevated Barium concentrations, which are characteristic of thermal water (Fig. 4c). The water of the Ranomaimbo lake was characterized by particularly high conductivity levels and a high concentration of dissolved solids (Fig. 4c), such as Natrium (157 mg/l) and Nitrate (20 mg/l). These values reflect the high levels of pollution that are often associated with urban settlements. Taken together, our findings suggest that marbled crayfish can tolerate substantial variation in ecological parameters.

**Fig. 5:**
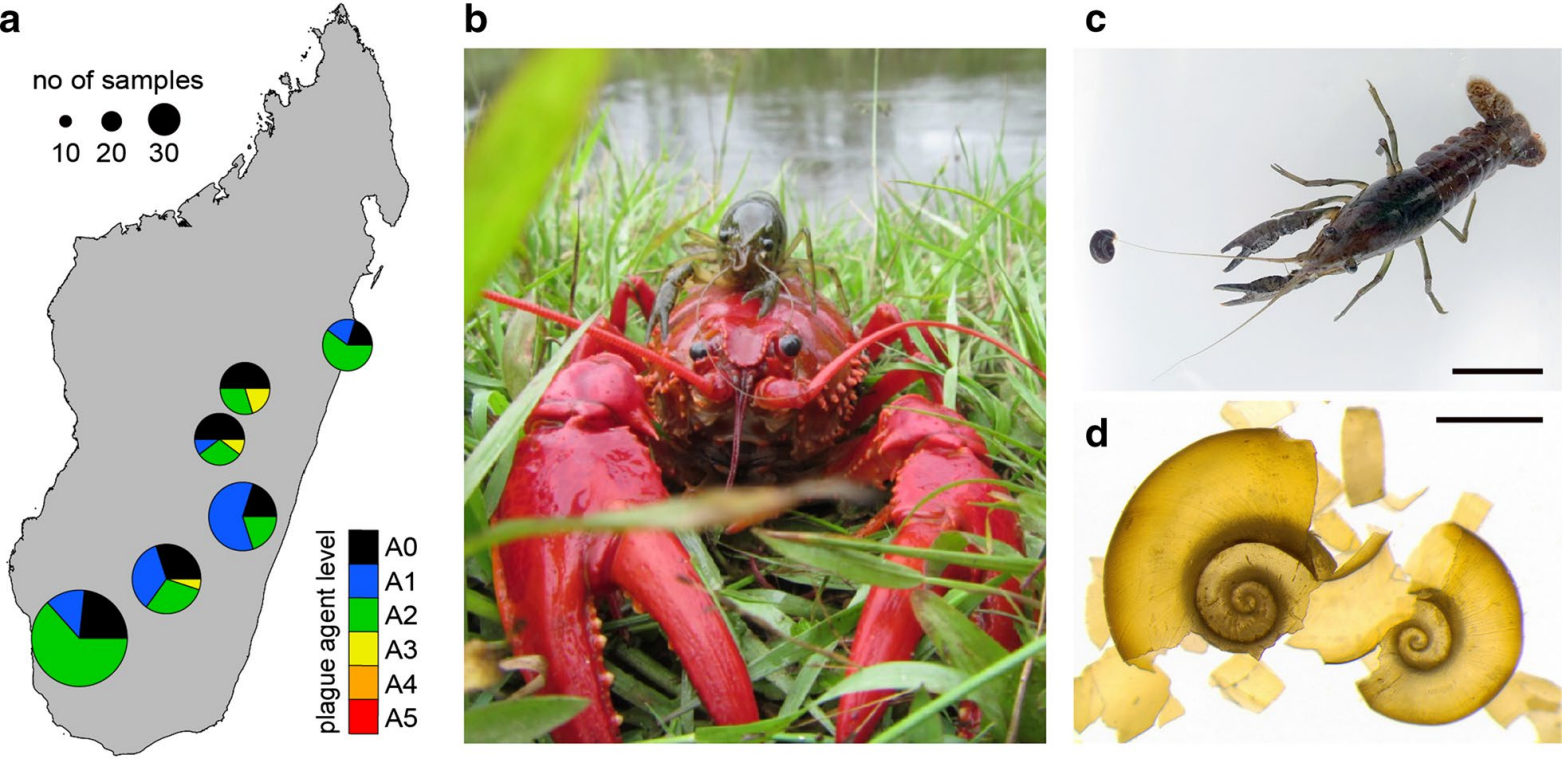
Biological impact of marbled crayfish on Madagascar. **a** Real-time PCR detection of the crayfish plague agent *Aphanomyces astacii* in animals that were collected in various locations in Madagascar. Numbers of animals analyzed per site are indicated by circle size (small = 10, intermediate = 20, large = 30). Agent levels are indicated by section colors. **b** Sympatry of marbled crayfish and *Astacoides betsileoensis* in the Andragnaroa river. **c** Laboratory experiment to test the predation of *Biomphalaria pfeifferi* snails by marbled crayfish. Bar: 2 cm. **d** Leftover *B. pfeifferi* shells, found after overnight co-housing with marbled crayfish. Bar: 2 mm. Map and pictures were produced by the first author

To explore the potential impact of these populations, we first investigated their infection status with the crayfish plague pathogen *A. astaci*. Indeed, a previous study had suggested low levels of *A. astaci* in marbled crayfish populations from Germany [22]. We investigated 100 animals from the five previously mentioned sites and from Antananarivo, where marbled crayfish were first detected on Madagascar [11]. Quantitative PCR (Fig. 5a) indicated undetectable (agent level A0 and A1) or very low (A2) levels of *A. astaci* DNA for the large majority (96/100) of samples. Only four samples presented with somewhat higher levels (A3). However, confirmatory sequencing and microsatellite analyses could not be carried out due to the low amounts of *A. astaci* DNA in the tissues. Notably, we also found marbled crayfish in natural habitats of two *Astacoides* species: in the Andragnaroa river, in sympatry with *A. betsileoensis* (Fig. 5b) and in a channel connected to a rice field in Sahavondronina, in sympatry with *A. granulimanus*. Marbled crayfish populations in those two locations have been known for at least 2 years, with no indications for crayfish plague outbreaks. Taken together, these results suggest that marbled crayfish do not trigger crayfish plague outbreaks in Madagascar.

We also noticed that the distribution area of marbled crayfish showed a strong overlap with the freshwater snail *Biomphalaria pfeifferi*, which acts as the main intermediate host of the parasitic flatworm *Schistosoma mansoni* in Madagascar [27, 28]. However, we could not find *B. pfeifferi* at the locations that we analyzed for marbled crayfish, suggesting possible predation. To confirm this possibility, we performed a laboratory experiment by placing snails of different sizes in laboratory boxes with a single marbled crayfish (Fig. 5c). After the first night, all (N = 28) snails had been eaten by the crayfish, and only leftover shells (Fig. 5d) were found in the boxes. This suggests that marbled crayfish can act as effective predators of *B. pfeifferi*.

**Fig. 6:**
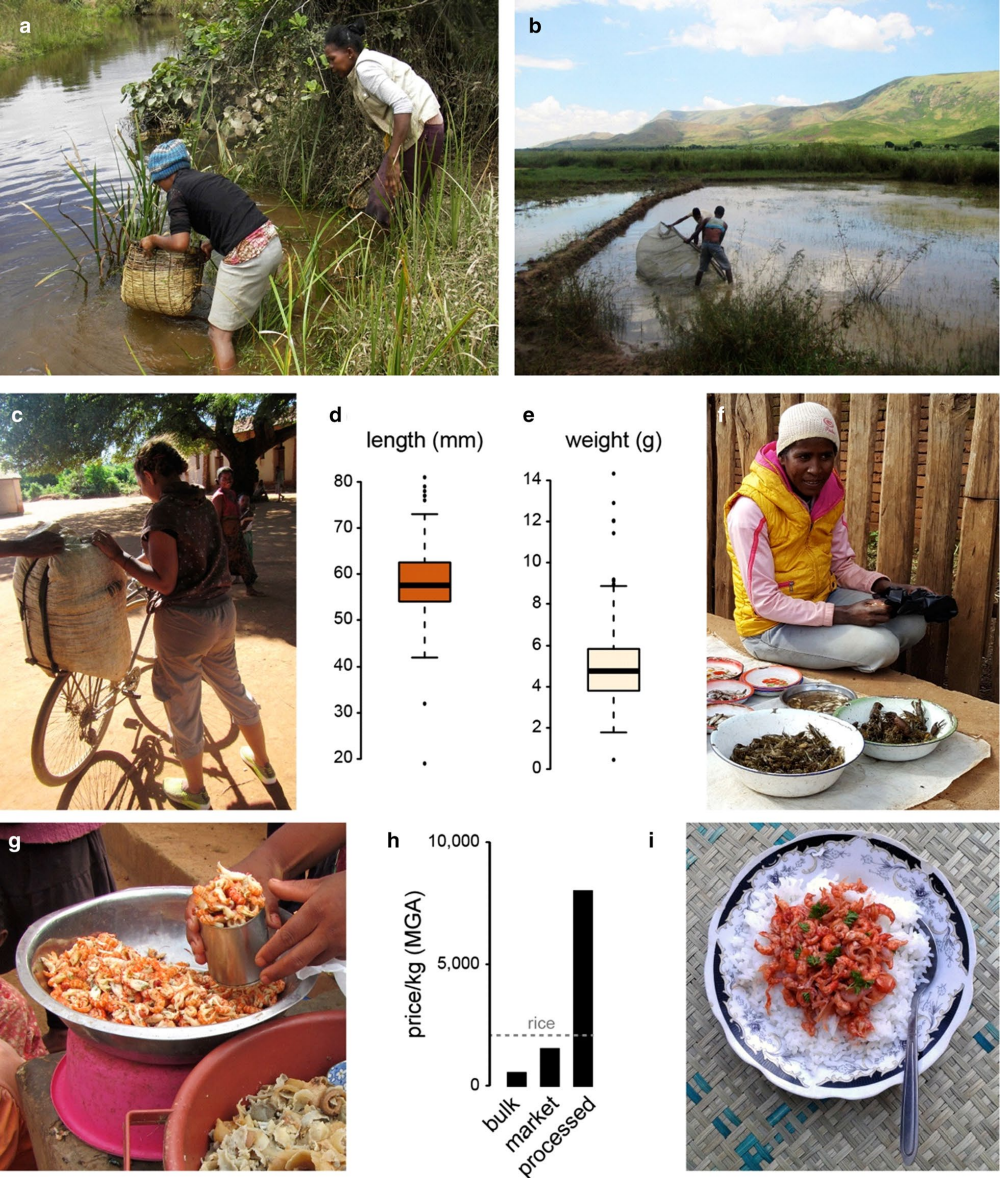
Commercial impact of marbled crayfish on Madagascar. **a** Marbled crayfish fishing in the Andragnaroa river, using the traditional Malagasy fishing tool (“tandroho”). **b** Farming on marbled crayfish on rice fields in Ihosy. **c** Commercial distribution of live marbled crayfish in large bags. **d** Length and **e** weight of 200 arbitrarily sampled animals from a commercial distribution bag. **f** Market sale of live marbled crayfish in Antsirabe. **g** Market sale of processed marbled crayfish tail meat in Antananarivo. **h** Representative prices (in MGA/kg) for various quantities and qualities of marbled crayfish. **i** Typical marbled crayfish-based food: rice with marbled crayfish in tomato sauce. All pictures were produced by the first author

Finally, we also addressed the emerging role of marbled crayfish as a food for human consumption. Marbled crayfish can be easily caught in rivers and ponds using traditional Malagasy fishing tools (Fig. 6a). Furthermore, the animals are also farmed in larger quantities on rice fields (Fig. 6b). For commercial distribution, 60–80 kg of live animals are packed in large bags (Fig. 6c) and then sold to consumers and/or local vendors. The measurement of 200 arbitrarily sampled animals from commercially distributed marbled crayfish established a median total length of 57 mm (Fig. 6d) and a median weight of 4.8 g (Fig. 6e), thus illustrating the commercial relevance of relatively small animals. Marbled crayfish currently represent an important component of the animal protein supply on local markets in all areas that were analyzed in this study and are being sold both as live animals (Fig. 6f) and as boiled and processed tail meat (Fig. 6g). Prices ranged from 500 to 1500 MGA per kg for live animals to 8000 MGA per kg for tail meat (Fig. 6g), which is comparable to the price of rice (approximately 2000 MGA per kg, Fig. 6g). Popular marbled crayfish foods include deep-fried beignets and rice with marbled crayfish in tomato sauce (Fig. 6h). The increasing acceptance and popularity of marbled crayfish foods in Madagascar is likely to further increase their commercial demand and intentional propagation.

## Discussion

Crayfish distribution and density is often influenced by specific habitat requirements [29, 30]. However, some crayfish species, such as *Procambarus clarkii*, are known to tolerate a broader range of environmental factors, such as water temperature, oxygen level and/or aquatic ecosystem type [31, 32]. Another example is the mother species of the marbled crayfish, *P. fallax*, which has been shown to inhabit aquatic ecosystems with various trophic levels, pH levels and water temperatures (summarized in [33]). Our findings are consistent with the notion that the marbled crayfish can tolerate a broad range of ecological parameters [33]. Furthermore, its successful colonization of diverse habitats in Madagascar clearly suggests high plasticity towards physico-chemical parameters and diverse biocenoses. Additionally, we have shown that altitude, which is closely linked to the diverse bioclimatic zones in Madagascar, has a negligible impact on marbled crayfish distribution. These findings significantly expand the suitable habitat of the global marbled crayfish population [21].

Adaptation to new environments is often explained by the selection of genetic variants. Interestingly, however, marbled crayfish are genetically identical, which suggests a central role of epigenetic mechanisms, such as DNA methylation, in their adaptation [8]. A recent study has provided a detailed characterization of DNA methylation patterns in marbled crayfish [34]. Interestingly, more than 2000 genes were found to be less methylated in marbled crayfish than in *P. fallax*, which was associated with more variable gene expression in marbled crayfish [34]. Gene expression variability has been suggested to facilitate adaptation in corals [35], but a functional role of DNA methylation in the adaptation of marbled crayfish remains to be shown.

Our study provides further insight into the impact of marbled crayfish in Madagascar. Traditionally, the impact of invasive crayfish species is defined by their disruptive effects on ecosystems and their capacity to transmit pathogens to naive hosts [36]. Indeed, the invasive spread of infected marbled crayfish could potentially have catastrophic consequences on the seven endemic freshwater crayfish species of Madagascar. Encouragingly, we found no evidence for crayfish plague outbreaks. *A. astaci* levels were below the limit of detection in 96 of the 100 samples analyzed, with the remaining four samples showing very low levels of DNA that could not be confirmed by sequencing. Also, we have observed two of the seven endemic crayfish species in sympatry with marbled crayfish, and with no symptoms of the crayfish plague. These findings suggest that either the marbled crayfish in Madagascar do not transmit the crayfish plague agent or that the (as yet unidentified) *A. astaci* strain is not very virulent. It is also possible that *Astacoides* is resistant to the disease, similar the American crayfish species and the European freshwater crayfish *Autropotamobius pallipes* [37, 38]. It will be important to clarify the infection status of *Astacoides* in future studies.

Our results also shed light on the potential use of marbled crayfish as a biocontrol agent against *B. pfeifferi*, which functions as intermediate host of *Schistosoma* flatworms in Madagascar and other African countries [27, 39]. Schistosomiasis remains a major public health problem in Madagascar with infection rates exceeding 50% of the total population [40–42]. Several freshwater crustaceans have been suggested as biocontrol agents of mollusk intermediate hosts to limit or reduce schistosomiasis infestation [24, 43–45]. However, the distribution ranges of these species and *B. pfeifferi* show little overlap on Madagascar. In contrast, marbled crayfish are widely distributed in Madagascar and it will be important to analyze their impact on the local presence and population densities of *B. pfeifferi* and the prevalence of schistosomiasis.

After the first scientific survey in Madagascar in 2007–2008 identified marbled crayfish as an ecological threat [11], the Ministry of Agriculture, Livestock and Fisheries issued legislation to prohibit the transportation of live marbled crayfish [46]. In addition, it was claimed that marbled crayfish consumption might be unhealthy for humans. Together, these factors initially restricted the commercial distribution of the animals. However, freshwater crayfish are a popular food in Madagascar and the endemic species have been harvested and consumed in large quantities for a long time. While their fishing and farming is severely limited by their specific habitat requirements and slow growth [19], marbled crayfish can grow quickly in diverse aquatic ecosystems. Furthermore, marbled crayfish are easy to stock, as a single animal can give rise to a new population. As such, they represent an attractive source of income and a cheap alternative for nutritional protein. By now, marbled crayfish have become an abundant and popular food in their distribution area in Madagascar. We expect that this popularity will further increase their spread, similar to other invasive species that have extended their distribution range through commercial networks [47].

## Conclusions

The marbled crayfish is a newly emerging invasive species, but very little is known about its ecological interactions. Our study provides the first detailed description of habitats that were successfully colonized by marbled crayfish populations in Madagascar. Our findings suggest a substantial habitat diversity and thus convincingly establish the ecological plasticity of the animals. Our results also provide answers for additional, important questions. For example, we found no evidence for the transmission of the crayfish plague pathogen by marbled crayfish. We also show that the animals are active predators of the intermediate hosts for human schistosomiasis. Finally, we provide the first documentation for the commercial exploitation of marbled crayfish for human consumption. Altogether, our study thus identifies key factors for the ecological assessment of this new invasive species.

## Methods

### Field work

The study was carried out from October 2017 to March 2018 in Madagascar. Details about collection sites are provided in Additional file 1. Pictures of habitats were taken using a Canon PowerShot D30 digital camera or a Samsung Galaxy S6 camera. For each habitat, we chose sampling stations of 20 to 150 cm depth and 50 to 1000 cm width. Collections were done in the morning from 8:00 to 11:00 for 5 to 7 days. One effort unit is defined as 2 persons per site per hour on total surface area of 100 m^2^ represented either by a transect or a quadrat. Crayfish were caught without release, either by the traditional fishing tool “tandroho” (50 cm × 30 cm × 30 cm) or with a net (200 × 400 cm) or manually in burrows. Carapace length and total length were measured using a manual caliper and weight was recorded using a portable scale with 0.1 g precision. Female sex was morphologically confirmed by the presence of the gonopores on the base of the third pair of legs and the ovaries under the carapace. Abdominal musculature samples from three animals per site were preserved in ethanol for genotyping, while soft cuticles, uropods and legs were preserved for the molecular detection of the crayfish plague agent *Aphanomyces astaci*. After data collection, all animals were sacrificed according to current Malagasy legislation, which prohibits the release or live transportation of marbled crayfish. Statistical comparisons of body size structure among the sites were performed by the non-parametric test of Kruskal–Wallis. All maps were drawn with QGIS (Open Source Geospatial Foundation Project) version 2.18.7. The shapefiles used to draw the 5 bioclimatic boundaries [48] were downloaded from http://www.mobot.org/MOBOT/Research/madagascar/gazetteer/. Madagascar boundaries and altitudinal gradients shapefiles were downloaded from the Free Spatial Data in DIVA-GIS (http://www.diva-gis.org).

### Habitat parameters

For each sampling site, bottom sediments (mud, clay, sand) were visually identified and water temperature was recorded with a multiparameter device (Hanna Instruments HI991300) 10 cm below the surface between 8:00 and 10:00 in the morning. The same device was used to record the conductivity and the pH with a calibration at 25 °C. Neighboring vegetation and animals co-collected in the fishing tools were recorded for each site (Additional file 1). Finally, water samples were collected at each site, and stored in a cold and dark place. Barium, Natrium and Nitrate levels were determined by Raiffeisen-Laborservice (Ormont, Germany).

### Genetic authentication of marbled crayfish

Genomic DNA was isolated and purified from 100 mg abdominal musculature using a Tissue Ruptor (Qiagen), followed by proteinase K digestion and ethanol precipitation. Genotyping was performed by sequencing of a PCR amplicon from the mitochondrial *cytochrome b* gene, as described before [8]. The complete sequencing results are provided in Additional file 2.

### Crayfish plague analysis

The crayfish tissue samples were dried and pulverized with a Tissue Lyser II (Qiagen, Germany). DNA was extracted with the E.Z.N.A. Insect DNA Kit (omega bio-tek, USA) according to the manufacturer’s instructions. The qPCR protocol to detect *A. astaci* was identical to the one used in [49] with an increased annealing temperature and decreased annealing time [50]. The results of the qPCR are categorized in semi-quantitative levels called agent levels, with increasing amounts of *A. astaci* DNA. Agent levels A0 and A1 are generally considered negative, while A3 to A7 are considered positive (A2 is considered ambiguous).

### *B. pfeifferi* predation

*Biomphalaria pfeifferi* snails (N = 28, 1–4 mm, a kind gift from Nelia Luviano and Christoph Grunau, University of Perpignan) were divided into seven groups of four and placed in closed laboratory boxes with a single marbled crayfish (TL 6–8 cm) that had been starved for 24 to 48 h. The boxes were checked every hour, left unattended overnight from 18:00, and checked again on the next morning at 9:00. Representative images were acquired on a Stereomicroscope (Olympus SZX10).

### Market and trade investigations

Marbled crayfish value on the market was evaluated among three main groups: chiefs of locations (n = 11), fishermen and/or crayfish collectors (n = 27) and local vendors (n = 17). Chiefs were interviewed first to evaluate the status of marbled crayfish in the area, such as fishing activities, locations of populations and markets. They also identified local collaborators (e.g. fishermen and/or crayfish collectors) that could provide further information about crayfish locations, farming and prices on the markets. Local vendors were interviewed about the main consumers and the prices of live and processed marbled crayfish.

## Additional files

**Additional file 1.** Detailed descriptions of marbled crayfish habitats, including coordinates.

**Additional file 2.** DNA sequencing data.
