# Supplementary material for: Ecological plasticity and commercial impact of invasive marbled crayfish populations in Madagascar
Source: BMC Ecol. 2019 Feb 6;19:8. doi: 10.1186/s12898-019-0224-1 (PMC6366054; doi:10.1186/s12898-019-0224-1)
Supplement: Supplementary file 1 — Additional file 1. Detailed descriptions of marbled crayfish habitats, including coordinates. [file 12898_2019_224_MOESM1_ESM.pdf]

Table S1. Overview of marbled crayfish populations analyzed.

Location	Site name	Coordinates	Habitat	Collection depth (cm)	Ground sediment
Tamatave	Ampasimpotsy	S18°09.925’ E49°21.925’	pond	50-120	mud
Antsirabe	Ranomaimbo	S19°52.226’ E47°01.791’	lake	50-100	mud
Fianarantsoa	Andragnaroa	S21°17.551’ E47°22.292’	river	50-100	mud
Ihosy	Ihosy	S22°22.512’ E46°06.016’	river	100-150	mud
Bezaha	Anjingilo	S23°30.674’ E44°29.658’	rice field	10-20	mud

Table S2. Marbled crayfish-associated vegetation.

Site name	Family	Genus	Common name
Ampasimpotsy	Poaceae	*Paspalum conjugatum*	Herbaceous grasses
	Pontederiaceae	*Salvinia* sp.	Macorphyte
Ranomaimbo	Poaceae	*Paspalum conjugatum*	Herbaceous grasses
		Oriza spp.	Rice
		Phragmites sp.	Phragmites
	Pontederiaceae	*Eichhornia* sp.	Macrophyte
Andraganroa	Poaceae	*Paspalum conjugatum*	Herbaceous grasses
		Oriza spp.	Rice
	Cyperaceae	*Cyperus latifolius*	Herbaceous grasses
Ihosy	Poaceae	*Paspalum conjugatum*	Herbaceous grasses
		Phragmites sp.	Phragmites
Anjingilo	Poaceae	*Paspalum conjugatum*	Herbaceous grasses
		Oriza spp.	Rice

Table S3. Marbled crayfish-associated fauna.

Site name	Family	Genus	Common name
Ampasimpotsy	Cichlidae	*Oreochromis* sp.	tilapia (fish)
	Channidae	*Channa striata*	snakehead fish
	Poeciliidae	*Gambusia* sp.	mosquitofish
	Odonata		dragonfly
Ranomaimbo	Cichlidae	*Oreochromis sp.*	tilapia (fish)
	Coleoptera		beetle
Andragnaroa	Potamonautidae	*Hydrotelphusa agilis*	crab
	Parastacidaea	*Astacoides betsiloensis* (red type)	crayfish
		Astacoides granulimanus	crayfish
		Xiphophorus hellerii	small fish
	Unidentified		tadpole
	Coleoptera, Hemiptera, Odonata		insects
Ihosy	Cyprinidae	*Carassius auratus*	goldfish
	Cichlidae	*Oreochromis niloticus.*	tilapia (fish)
	Poeciliidae	*Gambusia holbrooki*	mosquitofish
		Xiphophorus hellerii	small fish
	Thiaridae	*Melanoides* sp.	mollusc
	Ranidae	*Aglyptodactylus madagascariensis*	frog
	Unidentified		tadpole
	Atyidae	Unidentified	shrimp
	Potamonautidae	Unidentified	crab
	Coleptera, Hemiptera, Odonata		insects
Anjingilo	Cichlidae	*Oreochromis* sp.	(tilapia) fish
	Poeciliidae	*Gambusia holbrooki*	mosquitofish
	Unindentified		tadpole
	Coleoptera		beetle
