# Supplementary material for: Ecological plasticity and commercial impact of invasive marbled crayfish populations in Madagascar
Source: BMC Ecol. 2019 Feb 6;19:8. doi: 10.1186/s12898-019-0224-1 (PMC6366054; doi:10.1186/s12898-019-0224-1)
Supplement: Supplementary file 2 — Additional file 2. DNA sequencing data. [file 12898_2019_224_MOESM2_ESM.pdf]

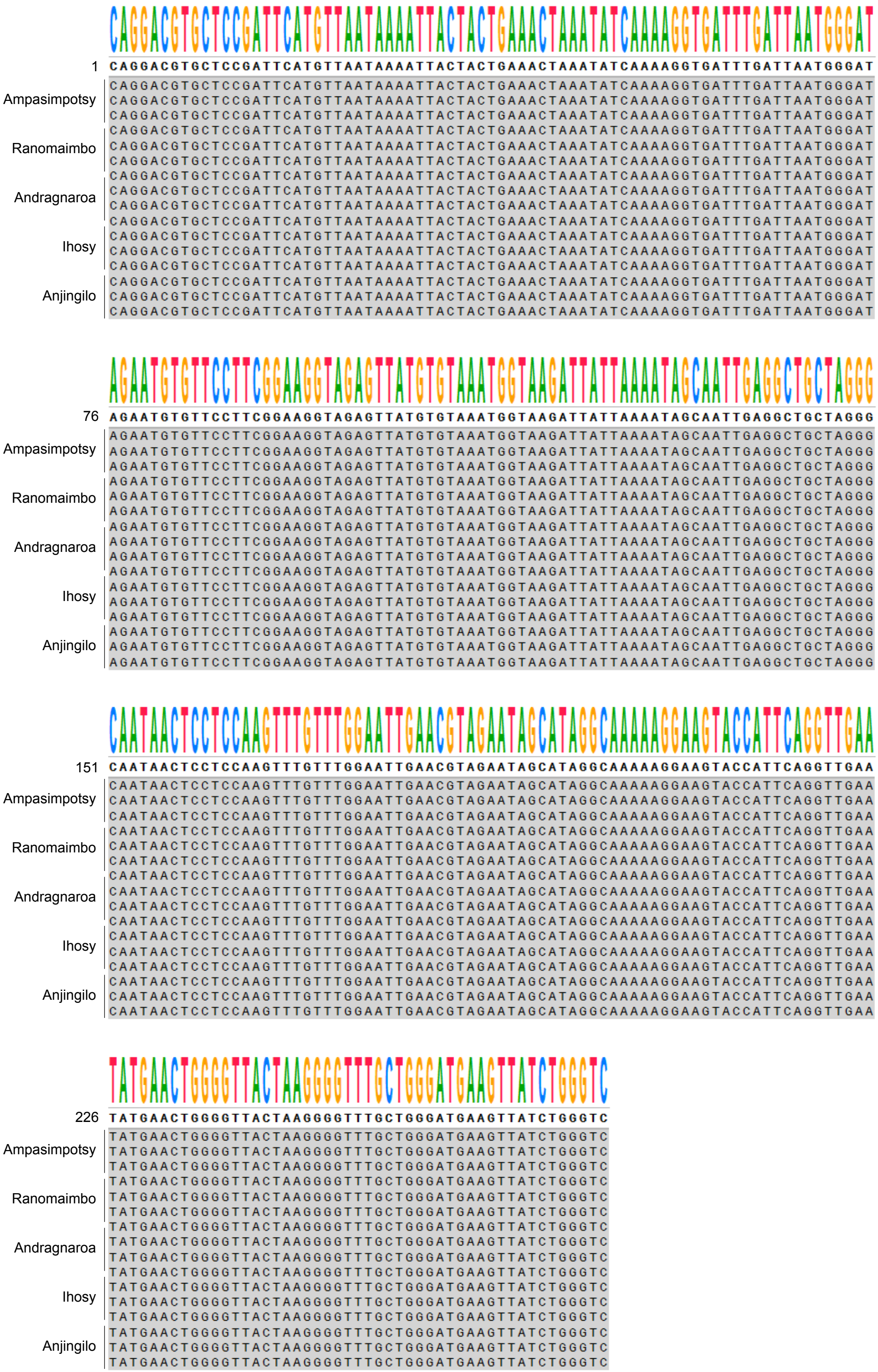

1 CAGGACGTGCTCCGATTCATGTTAATAAAATTACTACTGAAACTAAATATCAAAAGGTGATTTGATTAATGGGAT
Ampasimpotsy
Ranomaimbo
Andragnaroa
Ihosy
Anjingilo
CAGGACGTGCTCCGATTCATGTTAATAAAATTACTACTGAAACTAAATATCAAAAGGTGATTTGATTAATGGGAT
76 AGAATGTGTTCCTTCGGAAGGTAGAGTTATGTGTAAATGGTAAGATTATTAAAATAGCAATTGAGGCTGCTAGGG
Ampasimpotsy
Ranomaimbo
Andragnaroa
Ihosy
Anjingilo
AGAATGTGTTCCTTCGGAAGGTAGAGTTATGTGTAAATGGTAAGATTATTAAAATAGCAATTGAGGCTGCTAGGG
151 CAATAACTCCTCCAAGTTTGTTTGGAATTGAACGTAGAATAGCATAGGCAAAAAGGAAGTACCATTCAGGTTGAA
Ampasimpotsy
Ranomaimbo
Andragnaroa
Ihosy
Anjingilo
CAATAACTCCTCCAAGTTTGTTTGGAATTGAACGTAGAATAGCATAGGCAAAAAGGAAGTACCATTCAGGTTGAA
226 TATGAACTGGGGTTACTAAGGGGTTTGCTGGGATGAAGTTATCTGGGTC
Ampasimpotsy
Ranomaimbo
Andragnaroa
Ihosy
Anjingilo
TATGAACTGGGGTTACTAAGGGGTTTGCTGGGATGAAGTTATCTGGGTC
